# Oral vaccination with recombinant *Lactobacillus casei* expressing *Aeromonas hydrophila* Aha1 against *A. hydrophila* infections in common carps

**DOI:** 10.1080/21505594.2022.2063484

**Published:** 2022-05-01

**Authors:** Zelin Zhao, Hong Wang, Dongxing Zhang, Yongchao Guan, Shahrood Ahmad Siddiqui, Xiao Feng-Shan, Bo Cong

**Affiliations:** a College of Animal Science and Technology, Jilin Agricultural University, Changchun, Jilin, China; b Institute of special animal and plant sciences of CAAS, Changchun, Jilin, China

**Keywords:** *Lactobacillus casei*, immune response, *Aeromonas hydrophila*, common carp

## Abstract

The immunogenicity of Aha1, an OMP of *Aeromonas hydrophila* mediating the adhesion of bacteria onto the mucosal surface of hosts has been established. In this study, recombinant vectors, pPG1 and pPG2, carrying a 1366 bp DNA fragment that was responsible for encoding the 49 kDa Aha1 from *A. hydrophila* were constructed, respectively, then electroporated into a probiotic strain *Lactobacillus casei* CC16 separately to generate two types of recombinants, *L. casei-*pPG1-Aha1 (Lc-pPG1-Aha1) and *L. casei*-pPG2-Aha1 (Lc-pPG2-Aha1). Subsequently, these were orally administered into common carps to examine their immunogenicity. The expression and localization of the expressed Aha1 protein relative to the carrier *L. casei* was validated via Western blotting, flow cytometry, and immune fluorescence separately. The recombinant vaccines produced were shown high efficacies, stimulated higher level of antibodies and AKP, ACP, SOD, LZM, C3, C4 in serum in hosts. Immune-related gene expressions of cytokines including IL-10, IL-1β, TNF-α, IFN-γ in the livers, spleens, HK, and intestines were up-regulated significantly. Besides, a more potent phagocytosis response was observed in immunized fish, and higher survival rates were presented in *common carps* immunized with Lc-pPG1-Aha1 (60%) and Lc-pPG2-Aha1 (50%) after re-infection with virulent strain *A. hydrophila*. Moreover, the recombinant *L. casei* were shown a stronger propensity for survivability in the intestine in immunized fish. Taken together, the recombinant *L. casei* strains might be promising candidates for oral vaccination against *A. hydrophila* infections in common carps.

## Introduction

Bacterial infections are one of the main bottlenecks responsible for the immense economic losses in aquaculture industry worldwide [1], tail and fin rot, hemorrhagic septicemia and EUS caused by *Aeromonas* species account to 53% for the total infections in fish [2]. *Aeromonas*, a Gram-negative, facultative anaerobic bacterial species, is widely distributed in various aquatic environments, has long been documented as the causative agents for fish infections [3–5]. And *Aeromonas hydrophila* has been identified as the culprit of red sores disease in *Lateolabrax japonicus* and *Cyprinus carpio*; epigastric ulcerative syndrome in *Clarias garipenus* and *Eleginus gracilis* [6]. Human once eat contaminated food may develop enterogastritis and cutaneous infection or even systemetic infections, like peritoneal inflammation, microbiemia, cephalomeningitis, biliary diseases, HUS, and necrotic fasciitis [7].

Antibiotics used to be a prevailing option for treatments of *Aeromonas* infections in fish, which, however, posed increasing risks of antibiotic resistance to environments [8]. Vaccine has recently been valued as a superior alternative in the aquaculture industry due to its high efficacies and features that are safe to environment [9,10]. Currently, the established vaccine types consist of killed and live-attenuated vaccines, where effectiveness varies and the possibility of reversion to virulence in the case of live vaccines exists [11]. In addition, a range of subunit vaccines have been developed based on bacterial surface proteins presented on *A. hydrophila* including lipopolysaccharide, O-antigen, constitutively expressed single polar flagellum, iron-binding systems, and the bacterial cytotoxic enterotoxin (Act) [12]. Compared with the traditional killed and attenuated vaccines, these subunit vaccines appeared to be safer and induce more specific immune responses in host fish [13]. Unfortunately, none was commercially mature to date [14].

Bacterial adhesin (Aha1), an OMP of *A. hydrophila*, has been extensively studied for its immunogenicity and demonstrated capable of inducing both humoral and cellular immune responses in hosts to block bacteria from adhesion on the surface of hosts [15,16]. Its epitope exposed on the surface of bacteria which was considered to be responsible for its immunogenicity might be conserved across the genus *Aeromonas* [17]. Thus, vaccines developed on *Aeromonas* Aha1 are potentially able to protect against heterologous pathogens.

Mucosal surface was considered as the front-line defense, taking local MALT as a target to induce mucosal memory T and B cells generating local protection against invasive pathogens, mucosal immunization has therefore drawn a great attention recently [18]. Vaccine delivery strategies designed to deliver vaccines to the mucosal surface have become one of the research hotspots in this area. Optimal carriers should be easy to manipulate and low-cost. Attenuated pathogens used to be a favored option, capable of inducing mucosal immunity enhancing prevention of hosts against invading pathogens [19]. However, its unstable phenotypes usually lead to risks of restoring virulence, which constitutes a barrier for its applications. Nonpathogenic probiotic lactic acid bacteria (LAB), hence, became an ideal candidate for this delivery [20]. Long-term studies have shown that LAB offered several advantages, such as capable of adhering to epithelial cells allowing for persistent deliveries of biomolecules at mucosal sites; versatile for genetic manipulation; noninvasive; adaptability to harsh GIT milieu; and nonimmune tolerant ensuring it wouldn’t cause compromised immune responses in hosts [21].

Moreover, immunization methods are pivotal for valid immunoresponses. Traditionally, intramuscular or subcutaneous vaccination usually lead to failure or fairly weak mucosal immunity [22]. In contrast, vaccination via mucosal, such as nasal and oral, may induce decent immune responses [15]. Also, these are the initial infection sites, hence privileged for vaccination to neutralize pathogens at these entry points. Oral delivery outcompetes conventional routes due to its simpler administration, improved safety, wider and lasting immune responses [16]. Zhang *et al* [23]. revealed that OmpAI expressed by *L. casei* displayed significant immunity protection against *A. veronii* infections through oral administration in carp.

Herein, to reinforce immunoresponse in cyprinus carpio against *A. hydrophila* infection, the *Aha1* gene from *A. hydrophila* BSK-10 strain was cloned and expressed in *L. casei* CC16 strain. Common carp orally immunized with the recombinant *L. casei* generated significantly higher level of antibody in host fish. Our study offers a promising immunization method to defend cyprinus carpio against *A. hydrophila* infections, which might also be applicable to a broader perspective of aquacultural.

## Materials and methods

### Fish and ethical statement

Common carps (50 ± 0.1 g) were acquired from Fresh Water Fishery Research Institute of Jilin Province, PRC, kept in tanks at 25 ± 1 °C with 180 L flow-through waters. Fish were acclimatized for two weeks pre-experiment, fed with a commercial diet (Chen Hui Feed, China) two times daily with a feeding rate of 1% bodyweight. Every experiment procedure was completed as per the Guidelines of Animal Experiments of our university (JLAU08201409).

### Microbial strains, growth conditions, and plasmids

L.*casei* CC16 strain was separated from cheese, without plasmids, cultivated with MRS intermediary (Solarbio, PRC) under 30 °C free of agitation. *Escherichia coli* strain MC1061 containing the *Escherichia coli-lactobacillus* shuttle vector pPG was acquired from Guide Chem (Beijing, China), cultivated with LB broth under 37 °C, agitating at 180 rpm, added with Cm (Sigma, America) at an eventual content of 10 µg/ml. The *Escherichia coli-Lactobacillus* shuttle vector pPG-1 and pPG-2 were delineated in our previous Study [23]. pPG-1 is a surface-displaying plasmid comprising an anchor matrix-encoding pgsA gene derived from *hay bacillus*, located downstream targeted genes on the plasmid. Expressed protein of pPG-1 would be attached on the out surface of its carrier. Whilst, pPG-2 was a secretory plasmid, the expressed protein could be released into the bacterial surroundings. A ssUSP secretion signal peptide was inserted on backbones of both of the plasmids upstream target genes ensuring target proteins be secreted out of the membrane [24]. *Aeromonas hydrophila* BSK-10 was isolated from *crucian carp* (*Carassius carassius*), and thoroughly explored previously [25].

### Establishment of recombination Lactobacillus casei expressing Aha1 protein

The fragment of *Aha1* gene (1366 bp) of *Aeromonas hydrophila* BSK-10 (GenBank: EU518465.1) was amplified by PCR. Forward and reverse primers, P1 and P2 carried restriction sites *Bam*H I and *Xho* I; P3 and P4 carried *Eco*R I and *Sma* I, respectively, and these sites were underlined in the primer sequences (Table 1). The reactive process was completed under the status stated below: initial denaturalization under 94 °C for 300 s, followed by 30 cycles of thermal denaturalization under 94 °C for 60 s; annealing under 60 °C for 60 s; and extension under 72 °C for 60 s. The eventual extension reaction was completed for 600 s under 72 °C.

**Table 1.** Sequences and conditions of the primers used in PCR analysis

Prime	Sequence (5’-3’) Forward	Sequence (5’-3’) Reverse	Accession NO.
P1-2	CCGGATCCATGAAAAAGACAATTCT (*Bam*HI)	AACTCGAGTTAGAAGTTGTATTGCAGG (*Xho*I)	
P3-4	CCGAATTCATGAAAAAGACAATTCTG (*Eco*RI)	AACCCGGGTTAGAAGTTGTATTGCAGG (*Sma*I)	
IL-10	AACTGATGACCCGAATGGAAAC	CACCTTCTCCCAGTCGTCAAA	HQ250106.1
IL-1β	AACTGATGACCCGAATGGAAAC	CACCTTCTCCCAGTCGTCAAA	AJ249137.1
IFN-γ	AACAGTCGGGTGTCGCAAG	TCAGCAAACATACTCCCCAG	EU909368.1
TNF-α	TTATGTCGGTGCGGCCTTC	AGGTCTTTCCGTTGTCGCTTT	EU069818.1
β-action	CAAGATGATGGTGTGCCAAGTG	TCTGTCTCCGGCACGAAGTA	AB039726.2
dnaA	TCTGTTTATTTATGGTGGCG	ACTTTGAACTACTGGCGTC	
pPG1	AGGAGGAAGCTAGCACATGAAGAAA	ATGTAAAAATGGTTTAAAATATACT	
pPG2	AGGAGGAAGCTAGCACATGAAGAAA	GATCTCTCGAGTTCGAAGAGCTCAC	

**Figure 1.**
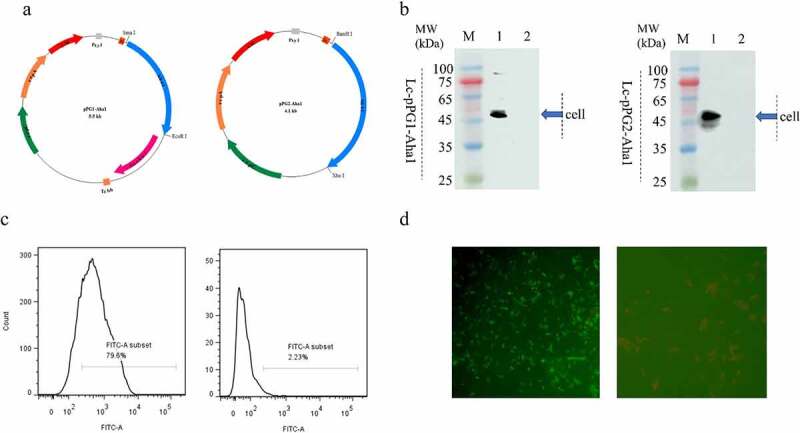
(a) Plasmid maps of the surface-displayed expressing Lc-pPG1-Aha1 (left), and secretion expressing plasmid Lc-pPG2-Aha1 (right). (b) Western blot analysis. Cellular extracts (Cell) of Lc-pPG1-Aha1 and Lc-pPG2-Aha1 were analyzed with western blotting. MW indicates the molecular mass markers (kDa). 1: Aha1 protein, 2: Ctrl. Blue arrows indicate Aha1 (49 kDa) secreted by Lc-pPG1-Aha1 in cell lysate (left) and Lc-pPG2-Aha1 in supernatant (right), respectively. (c) Aha1 antigen was detected on the surface of Lc-pPG1-Aha1 (left) by flow cytometry, but not on the surface of Lc-pPG (right). (d) Immunofluorescence microscopy analysis. Lc-pPG1-Aha1 (left) and Lc-pPG (right), magnification: ×1,000. There was green fluorescence on the surface of Lc-pPG1-Aha1 and no immunofluorescence reaction on the Lc-pPG cell surface.

The consequent PCR products were subjected to cleavage with *Sma* I/*Eco*R I and *Bam*H I/*Xho* I (TransGen, China), then cloned into the relevant sites of pPG expressing vectors to acquire recombination vector pPG1-Aha1 (anchor-type) and pPG2-Aha1(secretory-type) separately (Figure 1).

The transformation procedure was following previous descriptions [26]. Specifically, the two recombinant plasmids were initially constructed and propagated in *E. coli*, then were separately mixed with 100 μl of 1 × 10^8^ CFU/mL ice-cold competent *L. casei* CC16, and moved into a pre-cool Gene PulserTM one-off cuvette (interelectrode distance 0.2 cm) for electroporation with parameter of single pulse at 1.5 V, 25 F using an electroporator from Bio-Rad, America. Directly after the discharging, the suspension was desaturated in 900 µL of recovery medium (MRS broth with 0.3 M saccharose). After this, the microbes were cultivated for 3.5 h under 37 °C to recover and realize the expressing of antibiotics resistance, and afterward plated on MRS agar added with 10 μg/mL Cm.

Positive clones of pPG1-Aha1 and pPG2-Aha1 were validated via Sanger sequence identification (Comate Bioscience Co., Ltd. Jilin China). Meanwhile, *L. casei* transformed with empty pPG vector was utilized as the NC.

### Western blotting assay

The expression of the protein Aha1 was analyzed via WB [23]. The recombination strain *L. casei* 1 (Lc-pPG1-Aha1) and *L. casei* 2 (Lc-pPG2-Aha1) and control strain *L. casei* ctrl (Lc-pPG) were cultivated with MRS broth added with 10 µg/ml of Cm under 37 °C. 10 g/L of Xylose was supplemented into the bacterial suspensions to trigger protein expressing. Posterior to a 10-h cultivation, about 1 × 10^8^ CFU/mL cells were used for running SDS-PAGE, then moved onto a cellulose nitrate film (Baomanbio, PRC). The film was subjected to blockade in 5% skinmilk, afterward probed with mouse antiAha1 serum (Sino Biological, China) at 1:100 with PBS nightlong under 4 °C. Affinity-purified HRP-conjugated goat antimouse IgG (Sigma, America) was afterward utilized as the second antisubstance, cultivated under 4°C for 120 min according to the manufacturer’s instruction. Eventually, the visualization of the blots was realized via CLD with WB ECL substrate (Terom scientific) via an Amersham Image 600 (GE healthcare, the United Kingdom).

### Anchored protein expression of Aha1 on the surface of Lactobacillus casei detected by flow cytometry and indirect immunofluorescence assay

Recombinant strains, Lc-pPG1-Aha1 and Lc-pPG were induced by xylose, separately as described in 2.4. Approximately 1 × 10^8^ CFU of the induced bacterial cultures were subjected to resuspension in 50 μl PBS with 1% BSA and mouse anti-OmpAI antiserum (Sino Biological, China) at 1:100, then incubated for 60 min under 37 °C. Cells were then centrifuged under 5000 rpm for 20 min, followed with three washes with PBS containing BSA. After that, they were cultivated in darkness with FITC-conjugated goat antimouse IgG (Sigma, America) involving 1% Evans blue under 37 °C for 30 min. They were then cleaned three times in PBS containing BSA, and subjected to resuspension in 100 μL PBS in the absence of BSA. The fluorescence signal was detected by flow cytometry and analyzed by flow Jo software [27].

Immunofluorescent analyses were completed to identify the surface-displayed localization of the Aha1 proteins from Lc-pPG1-Aha1 as delineated in the past [28]. Briefly, 1 × 10^5^ cells of Lc-pPG1-Aha1 were subjected to resuspension in 50 µl PBS with 1% BSA and 100-fold diluted mouse anti-OmpAI antiserum, cultivated under 37 °C for 60 min. After that, they were cultivated with FITC-conjugated goat antimouse IgG (Sigma, America) involving 1% Evans blue under 37 °C for 120 min. The visualization of the cells was realized via a fluorescent microscopic device (Zeiss LSM710). The Lc-pPG was utilized as NC.

### Fish baits preparations

200 mL overnight bacterial suspensions of *Lactobacillus casei* 1 (Lc-pPG1-Aha1), *L. casei* 2 (Lc-pPG2-Aha1), and *L. casei* ctrl (Lc-pPG) in MRS broth added with 10 µg/mL Cm and 2% of xylose were blended completely with 20 g commercially available basal diet (Cheriser, Beijing, China), afterward desiccated in an oven under 40 ºC for 6 h. Feeds containing a mean of 1 × 10^9^ CFU/g of recombination *L. casei* were thus obtained. Fish in control group were fed with PBS-containing baits preserved under 4°C.

### Oral immunization

Totally 400 fish were employed in this study, divided equally into five groups (n = 80 per group): group *L. casei* 1 (Lc-pPG1-Aha1), group *L. casei* 2 (Lc-pPG2-Aha1), group *L. casei* ctrl (Lc-pPG), group *L. casei* CC16 and the negative control group PBS. Fish in each group were fed with bait which contained correspondent bacteria prepared in 2.6, respectively.

The fish were orally administered in three running days from day 1 to day 3. The reinforce immunization was implemented posterior to 14 days (i.e., day 18–20). On day 0, 7, 14, 21, 28 and 35 posteriors to the first immunization, 5 fish from every group were killed by overmuch anaesthetization via MS-222 (Sigma, America), respectively. The livers, spleens, HK, and intestines of the fish were quickly resected, placed in LN, and preserved under −80 °C, till later use.

### ELISA

The level of antibody induced by Aha1 was evaluated with ELISA according to a previous study [29]. Briefly, 96-well polystyrene microtitre dishes were initially coated with 5 μg/ml of purification Aha1 protein in 100 μl of coating buffering solution (15 mmol/L Na_2_CO_3_, 35 mmol/L NaHCO_3_, pH 9.5) under 4°C overnight. 2% BSA (in 1 × PBST) was then used to blocked nonspecific binding of the wells for 120 min under 37°C. The dishes were then cleaned twice in 1 × PBST, and 100 μl of serial diluted (1:50, 1:100, 1:200) fish sera separately in PBST were inoculated into each well in triplicate and cultivated for 60 min under 37 °C. The bound antisubstances were identified via HRP-conjugated anti-cyprinus carpio IgM (Stirling, Scotland) monoclone antibody diluted at 1:2000 for 60 min under 37 °C. After this, the reactive process was terminated by adding 50 μl of 1 N H_2_SO_4_. And afterward PNPP matrix (1 mg/ml) made with AP buffering solution (1 mM MgCl_2_, pH 9.8, 50 mM Na_2_CO_3_) was utilized for color development. Absorption was afterward identified at 450 nm via an ELISA analyzer (Tecan, America) [26]. End-point titers were expressed as the highest dilution yielding an OD ≥ 2 times higher in contrast to the average of the blank.

Besides, serum activities of SOD, AKP, ACP, LZM, and complement component C3 & C4 were examined via ELISA tools from Nanjing Jiancheng Bioengineering Institute, PRC, as per the supplier’s specification.

### Phagocytic activity of leukocyte

PBLs were separated as delineated in the past [28]. In short, 200 μl of blood drawn from tail vein of fish from every group was loaded into heparinized centrifugal tubes. 100 μl of 1 × 10^6^ cell/mL of *Staphylococcusaureus* bacterial suspension was inoculated into the blood, then incubated at 25 °C for 45 min, and shaken at 180 rpm every 10 min [30]. The supernate was then removed, the upper tier of centrifuged sediment was utilized to coat on a slide and desiccated in the atmosphere, then subjected to fixation with methyl alcohol and dyed in Giemsa liquor (Solarbio, PRC). 200 leukocytes were then randomly selected under 100x oil microscope to calculate the white blood cell PP and white blood cell PI were computed as follows: PP = white blood cells phagocytosing microbes/white blood cells × 100% PI = microbes phagocytosed by white blood cells/white blood cells phagocytosing microbes × 100% [31]. Each experiment was performed in triplicates and repeated three times.

### Survival of recombination Lactobacillus casei in the fish intestines

Two days after the prime immunization, three *cyprinus carpio* were randomly chosen from each group. All intestines were harvested and emulsified with PBS, and then serial-diluted (10^3^, 10^4^, 10^5^, 10^6^ -fold). To determine the survival of recombination *Lactobacillus casei* in the carp’s intestines [32, 33], the diluted mixtures were plated. Onto MRS agar plates supplemented with Cm, PCRs, and sanger sequencing were performed for verification of selected single colonies. Primers specific for pPG-1 and pPG-2 recombination vectors and the house-keeping gene dnaA of *Lactobacillus casei* were presented in Table 1.

### Genetic expression assay

Overall RNA from the tissues of livers, spleens, HK and intestines of the entire *common carps* tested was extracted with the kit of Simply Total RNA (Bioflux-Bioer, Hangzhou, China). cDNA was produced via the PrimeScript. RT reagent tool contained a gDNA eraser (Takara, PRC) to remove genomic DNA contamination in RNA samples. Gene expressions of IL-10, IL-1β, IFN-γ, and TNF-α in various tissues were evaluated by 7500 Real-Time PCR systems (Applied Biosystems®) via THUNDERBIRD SYBR qPCR Mix Kit (TOYOBO, China) in 96-well reactive dishes (AxyGen America). Every primer of immunity-associated genes utilized was on the foundation of previous publications and listed in Table 1. Two-step RT-qPCR were finished, of which all RT reactions and subsequent qPCRs were completed as per the supplier’s specification. Briefly, one qPCR reaction contained: 1 μl cDNA containing 1000 ng of reverse-transcribed RNAs, 12.5 μl 2 × SYBR Green Master Mix (Takara, Japan), forward and reverse primers (1 μL of each, 10 μL) and 9.5 μl ddH_2_O. The PCR conditions were as follows: 94°C for 30 s, 40 cycles of 94°C for 3 s, 60°C for 30 s. Every qPCR was completed for three times. RNA sample of day 0 was utilized as the experiment control, and the β-actin that encoded genes was utilized as the inner reference.

Each gene expression level was standardized with the expression of β-actin via the 2^−ΔΔCt^ approach [32]. The specificity of reactions was verified by analyzing melting curves. Data were analyzed by the strata gene MxPro software (stratagene m×3005p, USA).

### Challenge testing

After resuscitation, the vaccinated fish were intraperitoneally injected with 200 μl of 5 × 10^6^ CFU/ml (5 LD _does_) of *A. hydrophila* BSK-10 strain. After this, fish were monitored for the following 28 days, and the post-challenge survival rates (RPS ratios) were computed as per the equation below: RPS = {1 - (% death in inoculated fish/% death in control fish)} × 100. Every challenging test was performed three times.

### Statistics

All statistical analyses were performed using SPSS v.16.0 software and GraphPad PRISMM v7.0. One-way analysis of variance (ANOVA) followed by Tukey’s tests were performed to analyze differences between the treatments. *p* < 0.05 was considered as significant difference between groups. Data were presented as the mean ± standard deviation (SD).

## Result

### Recombinant Lactobacillus casei and expression of Aha1 protein

Two types of recombinant plasmids, anchored pPG1-Aha1 and secretory pPG2-Aha1, were constructed by cloning the gene *Aha1* of *A. hydrophila* into plasmid pPG612 in *E. coli* strain MC1061, subsequently, the *L. casei* recombinants were generated by introducing the two plasmids constructed into strain *L. casei* CC16 by electroporation, respectively (Figure 1(a)). Both of the recombination *Lactobacillus casei* expressed the anticipated Aha1 protein posterior to a 10-h cultivation with Xylose, which was validated via WB, in which a 49 kDa protein was identified in both cellular lysates of Lc-pPG1-Aha1 and Lc-pPG2-Aha1. Nevertheless, the control strain that carried empty pPG vector didn’t generate such protein (Figure 1(b)). These observations suggested that the Aha1 proteins were expressed and excreted by the *Lactobacillus casei* recombinants. Flow cytometry showed significant FITC fluorescence signals were present for strain Lc-pPG1-Aha1 in contrast to the NC strain, suggesting the presence of Aha1 on bacteria surfaces (Figure 1(c)), which were confirmed by the observation under a fluorescence microscopy, where fluorescence stained Aha1 protein was shown on the surface of Lc-pPG1-Aha1cells, rather than on that of the control cell (Figure 1(d)).

### Survival of recombination L. casei in the fish intestines

**Figure 2.**
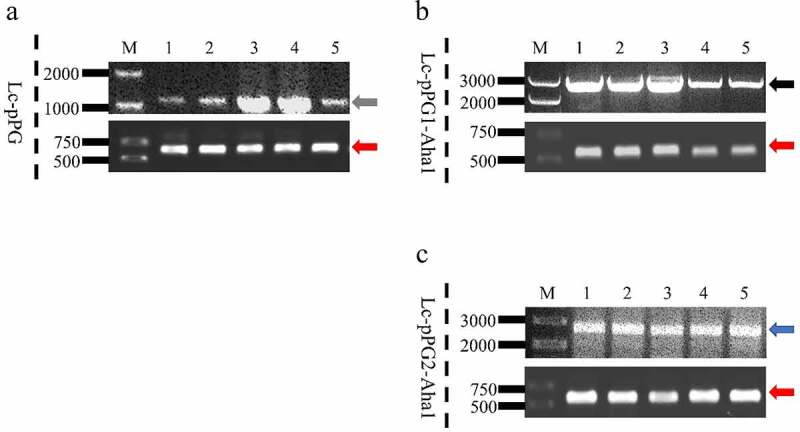
The colonies were directly PCR with pPG1 specific, pPG2 specific (above each group) or *L. casei* DnaA specific (below each group) primer pairs, and further analyzed by DNA sequencing, which was consistent with the speculated sequence. M: DNA ladder (bp), Lane 1-5: PCR products of recombinant *L. casei* with specific primers, gray (1250 bp), black (2392 bp), blue (2178 bp) and red arrow (615 bp).

The recombination *Lactobacillus casei* was recovered from the guts of immunized fish fasted for 2 running days, whereas rarely isolated in fish of the control group that were served with baits prepared with PBS. The plasmids extracted from the sub-cultured single colony were confirmed via PCR and sequencing, while the colony were verified as *L. casei* by examination of the housekeeping gene of *dnaA*. (Figure 2). Colony counts of the recombination *Lactobacillus casei* in the intestinal tract of the experiment group were shown remarkably greater in contrast to the controls (Table 2).

**Table 2.** The distribution of recombinant *L. casei* in the intestine

Group/Intestine	Population of recombinant lactic acid bacteria (CFU/ml)		
	Prosogaster	Mid-gut	Hind-gut
Lc-pPG1-Aha1	6.3 × 10^3^	1.6 × 10^5^	4.3 × 10^5^
Lc-pPG2-Aha1	4.8 × 10^3^	7.3 × 10^4^	2 × 10^5^
Lc-pPG	2.4 × 10^3^	6 × 10^3^	9.2 × 10^3^
PBS	-	-	-

### Immune responses induced in common carps

**Figure 3.**
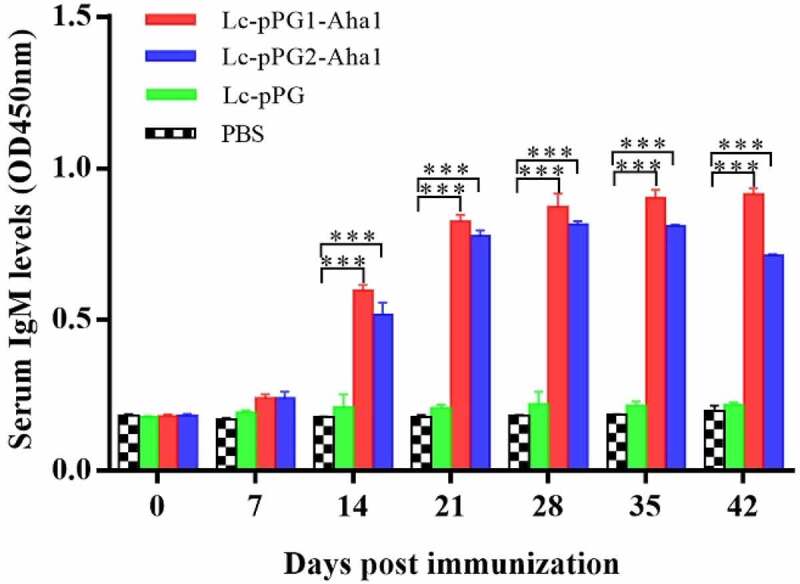
Relative content of the Aha1-specific IgM in serum (n = 5 fish/group) following treatment by Lc-pPG1-Aha1, Lc-pPG2-Aha1, Lc-pPG and PBS. Data are presented as mean SD fold increase relative to PBS control. *: *p* <0.05, **: *p* <0.01, ***: *p* <0.001.

Significantly improved humoral immunoresponses to the recombination *Lactobacillus casei* in experimental common carps after oral administration were revealed by ELISA. IgM content peaked at 0.833 μg/ml on the 25th day posterior to immunization, whereas didn’t trigger this response in fish of control group comparatively (Figure 3). In addition, averagely, fish exhibited a lagged reaction to secretion-type of recombinant protein in contrast to anchor-type. (Figure 3)

**Figure 4.**
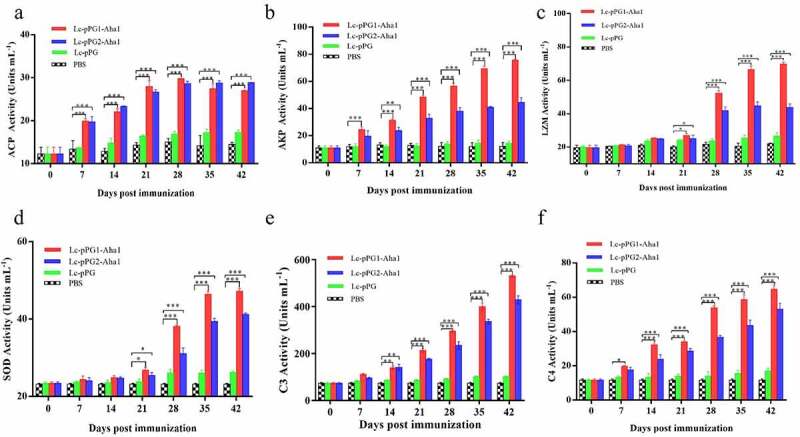
Humoral immune responses elicited by recombinant *L. casei*. Activities of acid phosphatase (ACP) (a) alkaline phosphatase (AKP) (b), lysozyme (LZM) (c), superoxide dismutase (SOD) (d), complement C3 (C3) (e) and complement C4 (C4) (f) in peripheral blood of common carp (n = 5 fish/group) after oral immunization. Data are presented as mean ± SD fold increase relative to PBS control. *: *p* <0.05, **: *p* <0.01, ***: *p* <0.001.

In addition, further evaluation of serum activities of ACP, AKP, LZM, SOD, and competent C3 & C4 induced by the oral immunization with the two *L. casei* recombinants were performed by ELISA. As shown in Figure 4(a-f), all enzymatic levels in experimental groups (Lc-pPG1-Aha1 and Lc-pPG2-Aha1) were remarkably reinforced in contrast to those of control PBS group (p < 0.05), with varied durations to reach their apexes, ranging from 14 to 42 days, respectively.

### Leukocytes phagocytotic assay

**Figure 5.**
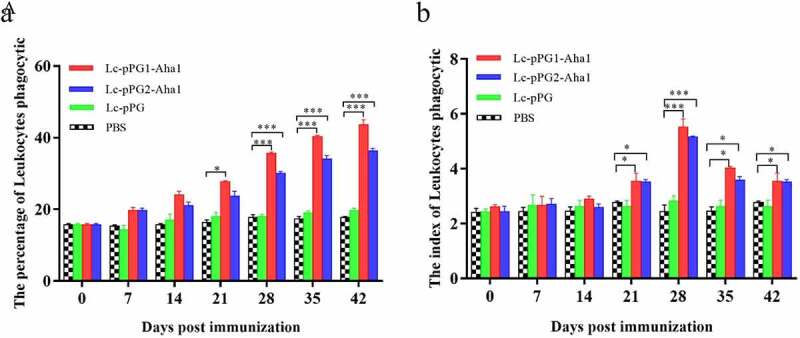
Leukocyte phagocytosis percentage and leukocyte phagocytic index in the serum (n = 5 fish/group) of fish vaccinated by Lc-pPG1-Aha1, Lc-pPG2-Aha1, Lc-pPG and PBS. Data are presented as mean ± SD fold increase relative to PBS control. *: *p* <0.05, **: *p* <0.01, ***: *p* <0.001.

As presented by Figure 5, leukocytes phagocytic ability of fish serum exhibited elevated tendencies after the fish were treated with the *L. casei* recombinants generated. PP and PI of leukocyte cells in fish of groups Lc-pPG1-Aha1 and Lc-pPG2-Aha1 were remarkably enhanced 14 days after immunization (*p* < 0.05) (Figure 5(a-b)), then maintained at high levels till reached the peak on day 25, which appeared to be dramatically greater in contrast to the PBS group (*p* < 0.05). Comparatively, these two parameters failed to show increases in the fish of Lc-pPG group (*p* > 0.05).

### Expression of immunity-associated genes in different organs

**Figure 6.**
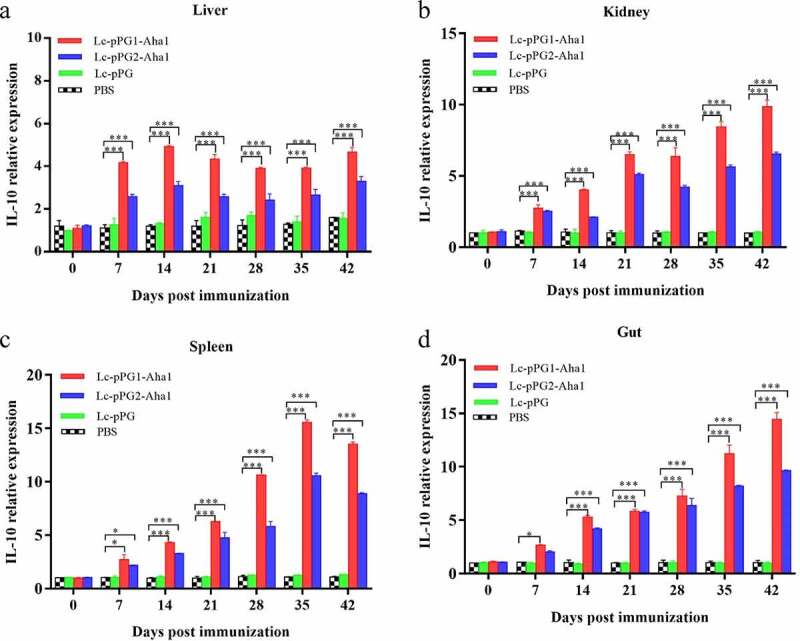
qRT-PCR analysis of the expression of IL-10 in Liver (A), Spleen(B),Kidney (C) and Intestine (D) of common carp (n = 5 fish/group) after immunization. Data are presented as mean ± SD fold increase relative to PBScontrol. *: *p* <0.05, **:*p* <0.01, ***: *p* <0.001.

**Figure 7.**
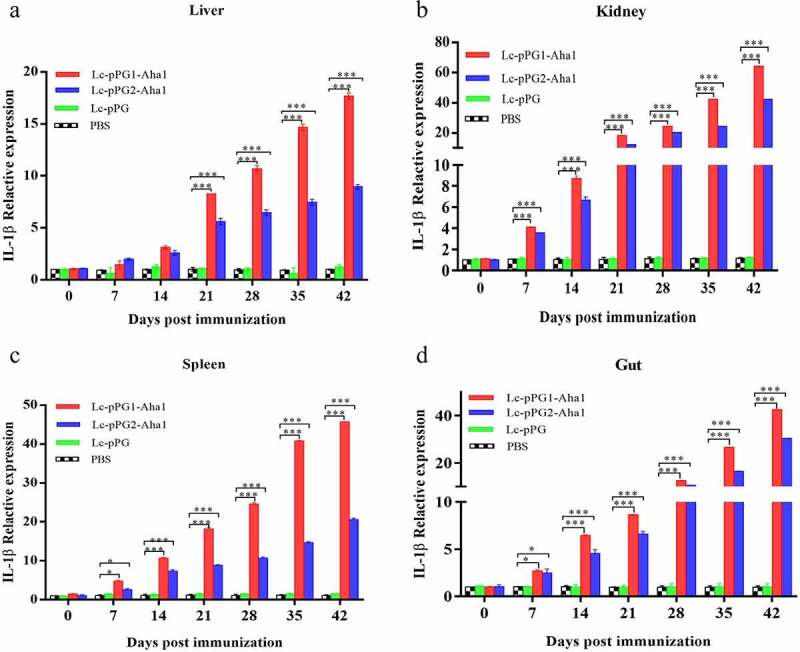
qRT-PCR analysis of the expression of IL-1β in Liver (a), Spleen(b), Kidney (c) and Intestine (d) of common carp (n = 5 fish/group) after immunization. Data are presented as mean ± SD fold increase relative to PBS control. *: p < 0.05, **: p < 0.01, ***: p < 0.001.

**Figure 8.**
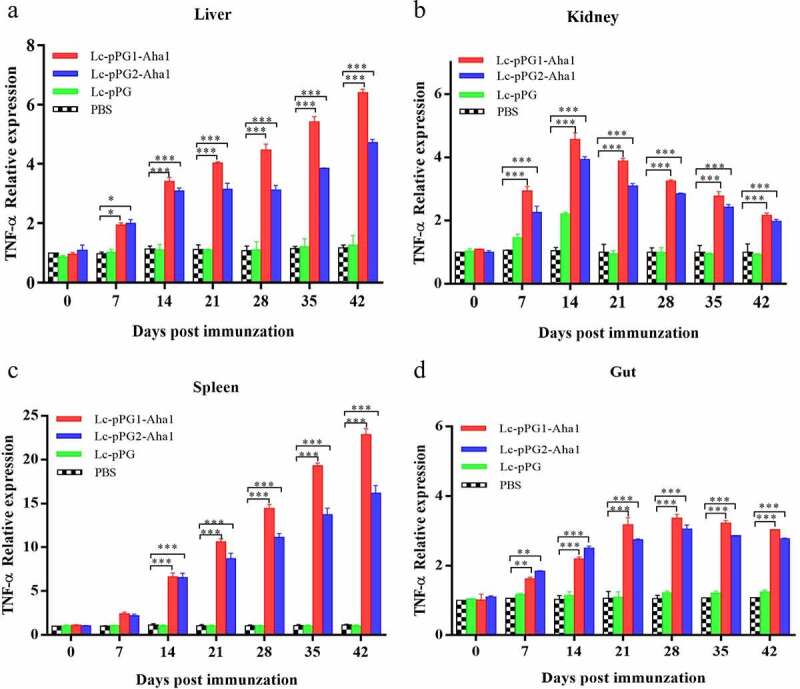
qRT-PCR analysis of the expression of TNF-α in Liver (a), Spleen(b), Kidney (c) and Intestine d) of common carp (n = 5 fish/group) after immunization. Data are presented as mean ± SD fold increase relative to PBS control. *: p < 0.05, **: p < 0.01, ***: p < 0.001.

**Figure 9.**
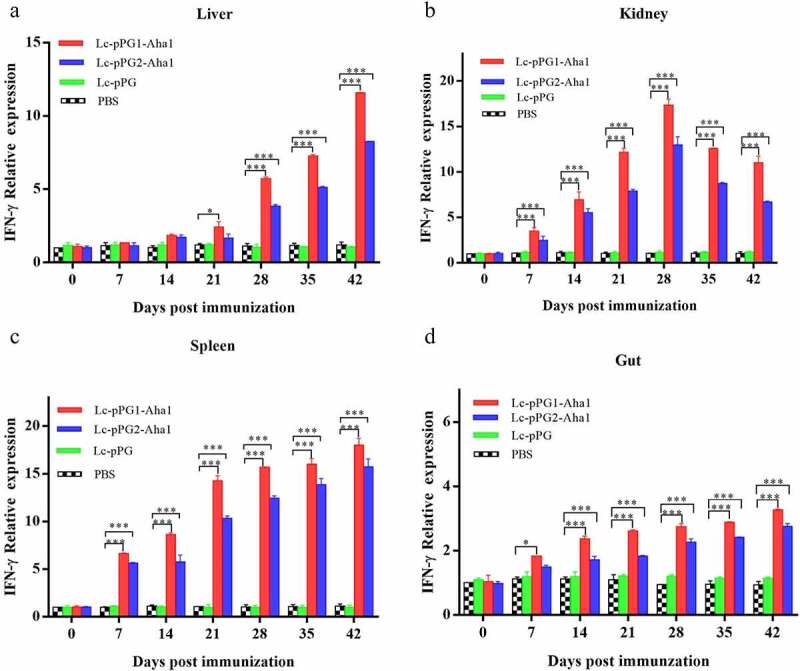
qRT-PCR analysis of the expression of TNF-α in Liver (a), Spleen(b), Kidney (c) and Intestine (D) of common carp (n = 5 fish/group) after immunization. Data are presented as mean ± SD fold increase relative to PBS control. *: p < 0.05, **: p < 0.01, ***: p < 0.001.

Gene expressions of IL-10, TNF-α and IFN-γ were all significantly upregulated within 14 days after the carps were immunized (Figure 6). However, gene expression of IL-1β between fish in both experimental recombinant groups didn’t show alterations within the initial 14 days after immunization in spleen and kidney, whereas significant upregulation was observed on day 35 after the booster immunization (Figure 7) (*p* < 0.05). The greatest genetic expression of TNF-α was presented in kidneys, in which it peaked on the 34th day (Figure 8). After seven days of immunization, the IFN-γ expressing in spleens, kidneys, and guts of Lc-pPG1-Aha1 group was significantly increased, reached the maximum on day 28 after the booster, and its expression in spleen reached the maximum on day 42 (Figure 9).

### Protection against the challenge of Aeromonas hydrophila

**Figure 10.**
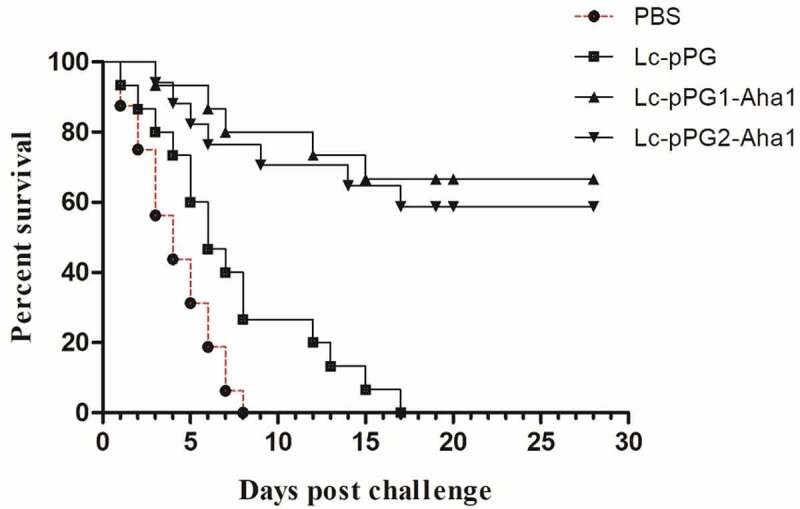
Survival rate of fish immunized with Lc-pPG1-Aha1, Lc-pPG2-Aha1, Lc-pPG, or PBS following challenge with the A.Hydrophila BSK-10 strain on post-immunization. 30 fish/group were used to record percent survival for 28 days.

To evaluate the protective effects induced by the vaccination of the recombination *Lactobacillus casei*, the immunized fish were infected with the strain *A. hydrophila* BSK-10 intraperitoneally with a lethal dose of 1 × 10^6^ CFU 34 days after immunization. The death rate was then documented for a successive 28-day period. Fish immunized with Lc-pPG1-Aha1 and Lc-pPG2-Aha1 displayed 60% and 50.0% survival rates respectively, whereas relatively low survival rates were exhibited in fish of control groups (Figure 10).

## Discussion

*Aeromonas hydrophila* is becoming a primary pathogen responsible for the vast of aquaculture infections, which incur great economic losses annually [34]. Aha1, an adhesion protein of *A. hydrophila*, could facilitate the bacterium adhering to the matrix of hosts’ epithelial cells, further invading into the hosts, also has been evidenced having high immunogenicity [14]. Vaccination, to date, was considered to be an effective approach for the treatments against *A. hydrophila* infections [35]. Especially, oral vaccination was characterized as low cost, as well as effective mucosal immunogenicity without inducing stressful responses caused by injection [36]. Moreover, the probiotic characteristics of *lactobacillus* species, and the natural property of excreting proteins with adhesion detachment functions of the original *Lactobacillus casei* CC16, made it an optimal live carrier for oral vaccination [37]. Herein, two types of *Lactobacillus casei* recombinants that expressed Aha1 of *A. hydrophila* were constructed, and their respective protective efficacies in common carps were evaluated. Initially, flow cytometry and immunofluorescent assays revealed that Aah1 proteins were expressed on the surfaces of the recombination Lc-pPG1-Aha1. And, it was found that Lc-pPG1-Aha1 induced a higher IgM level in fish serum compared to that of Lc-pPG2-Aha1, which was consistent with the study of Ju *et al*. (2020) [38], illustrating that anchor Lc-MCS-Malt induced stronger humoral responses than secretary forms of recombinants.

Complement system is an important innate immunity against pathogens invasion by covalent labeling of foreign molecules by complement C3 and C4 [39]. Herein, the contents of complement C3 and C4 in the sera of immunized fish increased dramatically. Consistent with other studies, this might be resulted from oral administration of probiotics. Nayak *et al*. (2010) demonstrated that probiotics could enhance the natural complement activities of fish [40]. A number of probiotic diets were associated with the stimulation of fish complement system [41,42].

Serum enzymes, such as ACP, AKP, and SOD, were reported to involve in anti-inflammatory, bactericidal activities and dissolution in host fish, which could facilitate to promote immunogenic responses [43,44]. And lysozyme (LZM) played a crucial role in anti-virus infections in fish [45]. All these nonspecific defense activities were shown enhancement in recombinant *L. casei* immunized *common carps*, and the responses in fish of Lc-pPG1-Aha1 group were remarkably greater in contrast to the Lc-pPG2-Aha1 group. Our results was consistent with the study of Kong *et al*. [46] which found that adding *L. casei* to the diet of crucian carps nonspecifically improved the enzymatic activities of ACP, LZM, and SOD. We speculated that the anchored Aha1 protein expressed by Lc-pPG1-Aha1 might involve in both the bacterial colonization onto the intestinal mucosal cells and activation of the host’s innate immune system, which, together, may lead to greater immune responses than that triggered by Lc-pPG2-Aha1. Tian *et al.* [47] also found that anchored *L. casei* Lc-pPG1-FlaA could induce a higher level of immunity.

Usually, bactericidal effects of leukocytes and phagocytosis were exhibited positive correlations with immune levels of host fish [48]. Zheng *et al* [49]. showed that probiotics could help improve the peroxidase level and leukocyte phagocytosis of Japanese eel. And lysozyme was responsible for activating phagocytosis. Phagocytosis cells produced a high level of ROS and thus enhanced the ability of attacking invading pathogens, which also facilitate promote leukocyte migration and eliminate pathogenic bacteria [50–52]. In this study, both recombinant *L. casei* strains were shown effectively improve the phagocytic activities of leukocytes. The PP and PI of immunized carp were observed to significantly increase after 21 days of immunization.

Cytokines are important immune cell secretions mediating repair of damaged tissues and resistance of infections [53]. IL-1β and TNF-α play indispensable roles in early inflammatory events. TNF-α, produced from stimulated mononuclear macrophagus, involved in infection preventions, and promoting inflammation cell differentiation. IL-1β takes participate in autoimmune diseases [54]. IFN-γ is mainly generated by T cells and NK cells, involved with the activation of monocytes and macrophages and regulation of the immune system [55]. IL-10, mainly generated by mononuclear cells, NK cells, macrophagus, and DCs, is an anti-inflammatory cytokine [56]. In this study, RT-qPCR was used to detect the expressing of those immune related genes in various tissues. Fish in the recombination *Lactobacillus casei* groups presented higher expressing levels of IL-10 gene in spleens, kidneys, and intestines. A similar trend of IL-10 expressing was observed in the fish exposed to probiotics [57,58]. We speculated that the up-regulation of IL-10 could facilitate to mitigate inflammatory reactions and body damages. Gene expressions of proinflammation cell factors IL-1β, TNF-α, IFN-γ were significantly up-regulated in livers, spleens, kidneys, and intestines of the recombination *Lactobacillus casei* groups, similar to the results of Ju *et al*. [38], which showed that recombinant *L. casei* triggered early pro-inflammatory responses and improved resistance against pathogens from invading in fish. A study by Tania *et al* [59]. also found that *L. plantarum* could exhibit immunomodulatory activity by inducing the up-regulation of IL-1β and TNF-α genes in rainbow trout HK, and the high expression of IL-1β can have more downstream effects on the immune system of fish [58]. Besides, mice immunized with recombination *L. casei* improved the production of TNF-α and IFN-γ [60]. Combined with previous reports, recombinant *L. casei* enhanced the pro-inflammatory response of immunized fish.

The accessibility of recombination *Lactobacillus casei* to the intestinal tracts in the immunized *common carps* was examined. Plate counts and colony-direct PCRs showed that approximately equal amounts of Lc-pPG1-Aha1 and Lc-pPG2-Aha1 survived in intestine of fish in both experimental groups, suggesting an survivability of the recombinant *L. casei*, which was similar to our previous study [32].

Moreover, consistent with other studies [30,45,61], fish receiving recombinant *Lactobacillus* exhibited higher survival rates after re-infected with *A. hadrophila* BSK-10. We ascribed this to an activated immune system due to the oral vaccination of the recombinant *L. casei* Lc-pPG1-Aha1 was formed in fish, also, we inferred the recombinant *L. casei* could inhabit on the intestinal surface and produce inhibitory substances as a barrier to pathogens. Future research, we would explore optimal the recombinant lactic acid bacteria, adjuvants and immunization regime that facilitate to produce stronger immune effects in common carps.

## Conclusion

Our study showed enhanced immune responses of carps to the immunization with Aha1 of *A. hydrophila* delivered by *L. casei* CC16. After oral administration, the *Lactobacillus* recombinants were more inclined to adhere to the intestinal tract of fish, exhibited more protective effects against infections of *A. hydrophila*. Especially, immunization with recombinant *Lactobacillus casei* expressing Aha1 of *A. hydrophila* which was attached on the cell surface might be a promising vaccination strategy against *A. hydrophila* infections in Carp.

## Data Availability

The authors declare that all data supporting the findings of this study are available in the manuscript or are available from the corresponding author upon request.
